# Getting Your First Publication in Medical Education—Why? What? Where? How?

**DOI:** 10.1177/23821205241242217

**Published:** 2024-04-01

**Authors:** Owen W. Tomlinson

**Affiliations:** University of Exeter Medical School, Faculty of Health and Life Science, 3286 University of Exeter, Exeter, UK

**Keywords:** publishing, early career, process

## Abstract

The process of getting one’s work published is a major milestone for many in their early academic and clinical careers. However, this process can be confusing and overwhelming for many who have yet to publish themselves. There are differing motivators for publishing work in our early career stages, alongside considerations, such as what we publish, where we decide to submit work, and how we logistically undertake the submission process. This commentary provides a holistic overview for the early career medical educator, empowering them to take the bold steps toward “getting published.”

You should publish that!

A saying that is simultaneously exciting, as it is apprehensive. It validates your ideas and perspectives; peers and senior colleagues believe what you have to say warrants sharing with the wider community. It also triggers a thought (and logistical) process that can be confusing and isolating; one where everyone's experiences and journey are different due to the vastly different work we all undertake.

The road to publication is one that brings reward for many, but also anxiety about the process, particularly for those embarking on the publication journey for the first time. Herein, this commentary briefly describes key steps and considerations in “getting published,” noting *why, what, where,* and *how* we publish.

## Why Do We Publish?

We publish for multiple reasons, notably, the advancement of science, education, and clinical practice. We share knowledge with others that enhances our understanding of the natural world around us but also work that can improve the quality of education we deliver, as well as the professional care standards and treatment practices for the patients our teaching affects in turn.

We also publish for our own personal benefit. Intrinsic factors such as an inherent interest in the research itself,^1^ but also career development^1^ whereby having publications can enhance Specialty Training applications in the United Kingdom.^2^ Surveys of medical schools in the United Kingdom suggest 10% to 14% of medical students publish during the course of their degree programs, disseminating various types of outputs, with multiple facilitators and barriers to such publications.^1,3^

## What Do We Publish?

The publication takes many forms—original research studies presenting novel results from trials, experiments and audits (make sure it is ethically approved!),^4^ narrative and systematic reviews of literature, protocols for upcoming studies, case reports, opinions, and perspectives on clinical and educational practice, letters to editors discussing previous work, guidelines and advice on how to perform techniques and processes, and database and informatics articles, among others.^1,3^

Publication, and contribution to the wider clinical and educational community, is not confined to results from large clinical trials or grand multicenter analyses. If it is something that can educate others and change practice, even in the smallest of ways, it is a valid contribution.

Everyone has something to contribute, no matter how big or small.

It is also worth considering if (and how) publication efforts can be managed as a team. Frequently, research studies will be a collaborative process, including senior and junior staff, and reviews often require multiple “reviewers” to reduce bias.^5^ If collaborating, individuals should only be included if they reach criteria for being a coauthor from the International Committee of Medical Journal Editors (ICMJE)—someone who has substantially contributed to design, acquisition, analysis, or interpretation, as well as drafting, and reviewing, and agreeing to be accountable for the work.^6^

## Where Do We Publish?

There are many avenues for us to disseminate our work, although the majority will occur via peer-reviewed journals. This process of peer-review independently verifies and considers the submitted results and interpretations, ensuring only valid and suitable (to the scope of the journal) submissions are published. Once published, articles are indexed in bibliographic databases such as PubMed, Embase, and Web of Science, making them searchable by future researchers. The peer-review process can take some time, so many people will opt to upload provisional copies of their work to “preprint” servers (eg, medRxiv.org); online spaces where new findings can be quickly disseminated, under the acknowledged caveat that they have not been peer-reviewed. This latter choice was a very common option during the COVID-19 pandemic, when our knowledge and understanding was rapidly evolving.

Conferences are also avenues for the dissemination of work, although tend to prefer research results, or syntheses of data such as systematic reviews, rather than pure opinions and perspectives. The work presented at conferences tends to (but not always) be published as a supplement to the meeting in a “book of abstracts”—brief summaries of the work that succinctly describes methods and results. However, as a “publication,” abstracts may not be indexed in bibliographic databases and can be overlooked as “gray literature” when other researchers conduct searches of the existing evidence base.

We must also be aware of the existence of “predatory publishers”; illegitimate organizations that lack transparency and reporting quality, who may prey on junior clinicians, researchers, and educators, while charging large fees for submissions.^7^ Their existence erodes the credibility of the literature base, and because these studies often lack peer review and ethical oversight, can be of harm if inadvertently implemented into clinical and educational practice. To avoid this risk for junior researchers, checklists are available to identify whether a publisher is legitimate, or illegitimate.^8^

## How Do We Publish?

Constructing submissions and turning them into “full” papers can be a long process, and guidelines are available^9,10^ to offset the known barriers of loss of momentum and poor follow-through.^11^ This is where collaboration comes to fruition, as these tasks can be divided among a team to ensure accountability and progress.

As part of the writing process, one must decide where to submit our work for publication, and then ensure that our submissions are appropriately formatted and contain all the pertinent information required by a journal, as every publisher will have slightly different requirements.

*Do submissions adhere to the correct word count? Do they have the correct referencing style? Are figures and tables formatted correctly? Does the manuscript need to be anonymized prior to review?* Mundane tasks, perhaps—but necessary ones.

Once you are ready to submit to the journal, a cover letter will help explain to the journal editor why you are submitting your work to that particular journal. These letters should explain briefly what the submission is about (Is it research? What does it show?), and how it fits the scope of the journal, as well as what the readership will gain from it. By contextualizing this to the remits of the journal, it shows that this is a targeted and well-designed submission.

If all things go well, this work will be sent for peer review, where comments and questions will be asked of the work. Sometimes these comments can feel like they undermine your efforts, but rest assured, they are aimed to positively impact and improve the final publication. You can respond (politely!) to reviewers, and the editors will consider if any changes to the submission warrant its final publication.

If reviewers and journal editors agree that the work is ready for publication, you will receive that magic “*Congratulations*” email in your inbox; concluding the publishing journey.

## Time to Publish!

This article briefly describes and clarifies the journey to “getting published.” There are many steps to consider, but it is a process that can be navigated and one that you are ready to embark upon. Use your networks, peers, and supervisors as guidance and support, and soon proudly say “*I published that!.”*
